# Evaluating the performance of anchored hybrid enrichment at the tips of the tree of life: a phylogenetic analysis of Australian *Eugongylus* group scincid lizards

**DOI:** 10.1186/s12862-015-0318-0

**Published:** 2015-04-11

**Authors:** Matthew C Brandley, Jason G Bragg, Sonal Singhal, David G Chapple, Charlotte K Jennings, Alan R Lemmon, Emily Moriarty Lemmon, Michael B Thompson, Craig Moritz

**Affiliations:** School of Biological Sciences, Heydon-Laurence Building A08, University of Sydney, Sydney, NSW 2006 Australia; New York University - Sydney, The Rocks, NSW 2000 Australia; Research School of Biology and Centre for Biodiversity Analysis, The Australian National University, Canberra, ACT 0200 Australia; Museum of Vertebrate Zoology, University of California, 3101 Valley Life Sciences Building, Berkeley, CA 94720 USA; Department of Integrative Biology, University of California, 3060 Valley Life Sciences Building, Berkeley, CA 94720 USA; School of Biological Sciences, Monash University, Clayton, Melbourne, VIC 3800 Australia; Department of Scientific Computing, Florida State University, Dirac Science Library, Tallahassee, FL 32306 USA; Department of Biological Science, Florida State University, 319 Stadium Drive, PO Box 3064295, Tallahassee, FL 32306 USA; The Commonwealth Scientific and Industrial Research Organization Ecosystem Sciences Division, GPO Box 1700, Canberra, ACT 2601 Australia

**Keywords:** Anchored phylogenomics, Coalescence, Phylogeography, Skink, Transcriptome, Ultraconserved elements

## Abstract

**Background:**

High-throughput sequencing using targeted enrichment and transcriptomic methods enables rapid construction of phylogenomic data sets incorporating hundreds to thousands of loci. These advances have enabled access to an unprecedented amount of nucleotide sequence data, but they also pose new questions. Given that the loci targeted for enrichment are often highly conserved, how informative are they at different taxonomic scales, especially at the intraspecific/phylogeographic scale? We investigate this question using Australian scincid lizards in the *Eugongylus* group (Squamata: Scincidae). We sequenced 415 anchored hybrid enriched (AHE) loci for 43 individuals and mined 1650 exons (1648 loci) from transcriptomes (transcriptome mining) from 11 individuals, including multiple phylogeographic lineages within several species of *Carlia*, *Lampropholis*, and *Saproscincus* skinks. We assessed the phylogenetic information content of these loci at the intergeneric, interspecific, and phylogeographic scales. As a further test of the utility at the phylogeographic scale, we used the anchor hybrid enriched loci to infer lineage divergence parameters using coalescent models of isolation with migration.

**Results:**

Phylogenetic analyses of both data sets inferred very strongly supported trees at all taxonomic levels. Further, AHE loci yielded estimates of divergence times between closely related lineages that were broadly consistent with previous population-level analyses.

**Conclusions:**

Anchored-enriched loci are useful at the deep phylogeny and phylogeographic scales. Although overall phylogenetic support was high throughout the Australian *Eugongylus* group phylogeny, there were nonetheless some conflicting or unresolved relationships, especially regarding the placement of *Pseudemoia*, *Cryptoblepharus*, and the relationships amongst closely-related species of Tasmanian *Niveoscincus* skinks.

**Electronic supplementary material:**

The online version of this article (doi:10.1186/s12862-015-0318-0) contains supplementary material, which is available to authorized users.

## Background

With the increasing access to genomic data enabled by high-throughput sequencing (HTS), phylogeneticists are applying phylogenomics to resolve deeper nodes in the tree of life (e.g., [1-3]). When used along with some form of genome reduction, HTS enables analysis of hundreds to thousands of loci across highly divergent organisms. Especially promising approaches include comparative RNAseq (e.g., [4-6]) and targeted hybrid enrichment (also referred to as sequence capture) using conserved or highly conserved orthologous loci as targets [1,7-9]. To the extent that common target loci are employed across diverse groups, these enrichment approaches have the potential to produce highly connectable data across orthologous loci, as was the case for mtDNA and cpDNA in the past.

For molecular systematists working at the phylogeographic interface between population genetics and phylogenetics, the utility of hybrid enrichment methods is not immediately obvious. Several methods have been used to generate data at low-moderate scales of phylogenetic divergence, including amplicon sequencing [10-12], various RAD-type methods (e.g., [13-15]), and target enrichment either using custom PCR-baits [16] or custom exon-capture designed from *de-novo* assembled transcriptomes [8]. Each of these has disadvantages – amplicon sequencing and RAD sequencing often generate very patchy matrices of loci by individuals, especially as divergence increases, and custom exon-capture requires more up-front investment. Custom PCR-bait capture has great potential for projects requiring tens of loci, but is not well suited where researchers aim to sequence hundreds of loci to improve phylogenetic precision. Directly mining transcriptomes (transcriptome mining or TM hereafter) is another source of potentially thousands of homologous loci, but phylogenetic analyses of mRNAs may be misled by recombination, especially in genes with exons separated by long introns. Moreover, transcriptome sequencing also requires very high quality, RNA-preserved tissue, and it remains expensive to sequence hundreds of individuals. Indeed, all phylogenomic methods require significant financial resources, but when factoring in the costs of reagents, sequencing, and labor versus the number of nucleotides sequenced, target enrichment type methods are far less expensive than traditional PCR-based methods [9].

Target enrichment using conserved targets – either “ultraconserved” elements (UCEs [17]) or conserved exons (anchored hybrid enrichment, AHE [1]) – could well yield useful data for phylogeographic inference, including historical demography, species delimitation and inference of divergence processes. However, given that the targets themselves are evolutionarily conserved, divergences and phylogenetic information content within and across closely related lineages could be low. In general, capture efficiency, and hence coverage, is highest across the relatively conserved target areas and decreases into the flanking sequences (e.g. intergenic regions or introns) that are expected to contain the most variability at this scale [9,17]. Further, given their high level of conservation, purifying selection could result in strongly biased estimates of population diversity, and thus of phylogenetic estimates via species trees or divergence parameters [18,19]. On the other hand, with the exception of genes that are highly conserved or consistently under strong adaptive or balancing selection, these issues may be less significant for loci mined from transcriptomes.

That conserved locus hybrid-capture might nonetheless have utility at a phylogeographic scale is suggested by a recent analysis of population divergence across multiple avian taxa from the Amazon basin that used anchored enrichment of UCEs [17]. Similarly, AHE has provided good resolution of phylogeny and divergence histories across closely related species and lineages of chorus frogs (*Pseudacris*; Lemmon et al., unpub. data). However, using non-random sets of the genome for evolutionary inference has potential to bias estimates of divergence and historical demography because depending on how these loci are ascertained, they might show idiosyncratic patterns from the rest of the genome. For example, one recent study found that data from UCEs versus RAD-Seq yielded very different estimates of divergence history parameters among phylogeographic lineages [19].

Here we explore the information content of AHE loci at different taxonomic scales including across genera, congeneric species, and phylogeographic lineages of the Australian *Eugongylus* group skinks (Squamata: Scincidae). In doing so, we both infer phylogenies and divergence histories for this group of lizards. We also compare the performance of AHE to the only other current source of multi-locus data for the *Eugongylus* group: exons mined from transcriptomes.

The *Eugongylus* group is a species rich clade of lygosomine skinks that includes ~40 genera and 420 described species. The common ancestor of a clade consisting of 17 extant genera colonized Australia and adjacent landmasses (New Zealand, New Caledonia, South Pacific islands) from Asia in the Early to Middle Miocene (42–22 Mya [20-22]). Species from three of these genera – *Carlia*, *Lampropholis* and *Saproscincus* – have been subjects of detailed phylogeographic analyses in wet forests of eastern Australia [23-28] and of studies of the evolution of reproductive isolation among phylogeographic lineages [29]. Previous molecular phylogenetic analyses using relatively few loci have inferred strong support for relationships among species within these genera (e.g. [23,30]). However, relationships among genera in the *Eugongylus* group remain largely obscure and are evidently difficult to resolve using few loci (Shea et al., unpub. data). Moreover, *Niveoscincus* and *Pseudemoia* are viviparous, and species in these genera show a range of both placental complexity and variation in mother-to-embryo exchange [31-34]. Without a robust intergeneric phylogeny, it remains impossible to uncover the evolutionary processes that explain the diverse ecological, physiological, and reproductive traits among Australian *Eugongylus* group skinks.

## Methods

We constructed two multi-locus data sets using from data collected from different sequencing methods. The first data set consists of 415 orthologous genes sequenced for 43 taxa using anchored hybrid enrichment [9]. The second data set includes 1650 exons from 1648 orthologous genes for 11 taxa that were mined from RNA transcriptomes.

### Taxon sampling design

We evaluate the performance of AHE and TM at the intergeneric, interspecific, and intraspecific phylogenetic levels. Our nested sampling design includes 9 of 17 Australian *Eugongylus* group genera as well as two individuals per phylogeographic lineage for *Carlia rubrigularis*, *Lampropholis coggeri*, *La. robertsi*, and *Saproscincus basiliscus* (Additional file 1)*.* As outgroup taxa, we sequenced *Lerista bougainvillii* of the *Sphenomorphus* group clade of lygosomine skinks, which shares a most recent common ancestor with the *Eugongylus* group 64–100 million years ago [22]. We used a single outgroup because this is as required for STEAC analyses (see below), and this makes the results directly comparable across methods. Inclusion of all three outgroups in RAxML analyses of the concatenated data set does not affect support for relationships of the ingroup (not shown). The final AHE data set includes 42 ingroup samples, and the TM data set includes 10 ingroup samples and a single outgroup in each data set (*Le. bougainvillii*).

### Anchored enrichment data collection and processing

Data were collected following the methods of [9] through the Center for Anchored Phylogenomics at Florida State University. Briefly, each genomic DNA sample was sonicated to a fragment size of ~300-700 bp. Subsequently, library preparation and indexing were performed following a protocol modified from [35]. Indexed samples were then pooled at equal quantities (eight samples per pool), and enrichments were performed on each multi-sample pool using an Agilent Custom SureSelect kit, which contained probes designed for anchored loci from multiple vertebrate genomes (Vertebrate 1.0, described in [9]). After enrichment, the six reactions were pooled in equal quantities for sequencing on a single PE100 Illumina HiSeq2000 lane. Sequencing was performed at the QB3 Vincent J. Coates Sequencing Laboratory at the University of California, Berkeley.

Quality-filtered sequencing reads were processed following the methods described in [9]. In short, reads were demultiplexed (with no mismatches tolerated) and pairs were merged following [36]. Merged reads were then scanned for matches to the probe region sequences of *Anolis carolinensis* using the high sensitivity approach described in [9]. Reads matching to a reference sequence for each individual were then aligned to produce an assembly for each locus. The reads were aligned using a script written by ARL that: (1) sorts the reads by number of matches to the reference, (2) for each read in the sorted list (starting with best-matched read), the position maximizing the match to the previous read in the list is noted, (3) if the best matching position does not generate a 90% match (of at least 20 bp) the read is skipped, (4) the entire sorted list is repeatedly traversed until no additional reads have aligned during a traversal (source code for all analyses in this study is available on Dryad doi:10.5061/dryad.g4mj2).

After assemblies were complete, consensus bases were called as follows: (1) all sites with less than 3-fold coverage and variant sites with less than 10-fold coverage were called as “N”, (2) invariant sites with coverage between 3-fold and 10-fold were called with the observed nucleotide, (3) for sites with greater than 9-fold coverage, the most common base was called unless the distribution of observed bases was unlikely to have arisen under a two allele model with equal allele frequencies. This likelihood was approximated using the equation p = (1-pbinom(nMax,n,0.5)), with n equaling the number of unambiguous base calls and nMax equaling the abundance of the most common base call. The most common base observed was called unless p > 0.05, suggesting that the two allele model could explain the diversity observed. In that case, an ambiguity code corresponding to all of the observed bases at that site was used in the consensus sequence. Sequences for each locus were aligned across species using Muscle version 3.8.31 under default settings [37]. Prior to phylogenetic analysis, each locus data set was trimmed to the second shortest sequence, and all gapped characters were removed.

### Transcriptome assembly, identification, and mining

Methods for sequencing, assembly, and identification of the transcriptomes of *Le. bougainvillii*, *Pseudemoia entrecasteauxii*, *P. pagenstecheri*, and *P. spenceri* contigs were similar to [38]. Briefly, approximately 5 gigabases of 100 bp paired-end reads were generated using Illumina HiSeq 2000 from pregnant uterine (*Le. bougainvillii*, *P. entrecasteauxii*, and *P. pagenstecheri*) or brain tissue (*P. spenceri*) from a single individual of each species. Contigs were assembled for each species with Abyss v1.3.4 [39] using kmer=69, and otherwise default parameters. Contigs ≤ 100 bp were removed. To identify each contig, we used blastx to align them to the genomes of *Anolis* lizard, chicken, mouse, human, platypus, and wallaby genomes (Ensembl build 64).

For *Carlia, Lampropholis,* and *Saproscincus* species, data were collected and processed as described in [40]. Briefly, for each phylogeographic lineage, we collected 3.5 Gb of paired-end reads from each of five adults’ livers. Reads were trimmed and merged using FLASH v1.0.2 [41] and Trimmomatic v0.16 [42]. We then generated an assembly across all individuals for that species using Trinity r2012-01-25 [43], and then removed redundancy across these assemblies using a custom merge script using blat, cd-hit-est, and cap3 [44-46]. The final transcriptome data sets included eleven taxa including species of *Carlia*, *Lampropholis*, *Pseudemoia*, *Saproscincus*, and *Le. bougainvillii* (outgroup).

### Assessing phylogenetic history

To test the performance of both 415 AHE loci and 1650 TM loci, we performed phylogenetic analyses for different taxonomic levels including the (i) the full 43 sample data set (*Eugongylus* group + outgroup) to assess performance at the deep phylogenetic scale, and (ii) *Lampropholis* only and *Saproscincus* only data sets to test performance at the interspecific and phylogeographic scale. For each of the AHE loci and TM loci, we first calculated the most appropriate models of sequence evolution for each locus using MrAIC [47] choosing the model with the best AIC score amongst 24 possible models (GTR, SYM, HKY, K2P, F81, and JC69, with and without I and Γ parameters). We then estimated a maximum likelihood gene tree for each locus using PHYML [48] using the appropriate model selected by MrAIC.

We performed concatenated data and species tree phylogenetic analyses for the full taxon, *Lampropholis* + *S. basiliscus* N1 (outgroup), and *Saproscincus* + *La. coggeri* N1 (outgroup). Invariant loci were excluded and the final AHE data sets for the full taxon, *Lampropholis*, and *Saproscincus* data sets were 415, 414, and 412 loci, respectively. There are no invariant TM loci in all three data sets and the full 1650 loci were used. Maximum likelihood phylogenetic analyses of the concatenated data sets were performed using RAxML [49] assuming separate GTR + Γ models for each locus. We then estimated clade support using 1000 pseudoreplicates of bootstrap resampling with the same partition and model scheme as the single-tree analyses.

Simulated and empirical analyses have shown that, if there is strong incongruence amongst loci, analyses of concatenated data sets may infer a strongly supported, but incorrect trees [50-52]. We therefore performed additional species tree analyses using STEAC [53]. STEAC estimates a phylogeny by calculating the average coalescence times across multiple loci. The method is useful because it is computationally tractable with large multi-locus data sets. On the other hand, it assumes incongruence between the gene and species trees is due only to incomplete lineage sorting rather than other phenomena such as hybridization.

STEAC assumes that gene trees are estimated without error, so to incorporate estimation error for individual gene trees in our STEAC analyses, we used 1000 bootstrap trees estimated per locus rather than a single point estimate tree. Although our phylogenetic analyses of concatenated data used RAxML, we chose PHYML [48], an alternative maximum likelihood phylogenetic program, for this analysis to enable efficient scripting of the STEAC analytical pipeline. We first calculated the most appropriate models of sequence evolution for each locus using the same procedure as above. For each locus, we performed a 1000 pseudoreplicate bootstrap analysis using these chosen models. We used STEAC to infer a species tree. We estimated support for this tree by calculating 1000 ‘bootstrap’ STEAC species trees, where each ‘bootstrap’ species trees draws (without replacement) one of the PHYML bootstrap trees for each locus (e.g., making use, in total, of 415,000 trees for the full AHE data set). Species tree analyses were performed using the phybase package in R [54]. Given the number of taxa and loci, use of Bayesian concordance analyses (e.g., BUCKy [55]) were not computationally feasible.

To evaluate the spatial distribution of variation across loci, and to enable comparison with results for ultraconserved elements (UCEs) [17], we assessed the distribution of phylogenetically informative characters (PICs)/across the 415 AHE loci. All loci were aligned so that the 0th nucleotide corresponded to the middle of the aligned locus. Moving laterally along the loci in both directions, we plotted the number of total loci (max = 415) with a PIC site at that nucleotide position across all taxa. For example, if there were 69 loci with a PIC at the 4th nucleotide position, then we recorded a value of 69. Because the loci vary in length, we overlaid these plots on a distribution of locus lengths calculated by counting the number of loci with any character at a nucleotide position. Unlike the alignments used for the phylogenetic analyses, we did not remove gapped characters to avoid artificially increasing the number of PICs near the alignment origin due to shortening the alignments. We repeated these analyses using only the *Lampropholis* and *Saproscincus* data sets. Finally, we constructed a “normalized” plot for the full-taxon data set by dividing the number of PICs at each nucleotide position by the number of alignments of that specific length.

### Inferring divergence histories

To further explore the utility of AHE markers for evolutionary inference, we inferred divergence history parameters for the five lineage-pairs for which we had prior estimates of divergence history from population samples of transcriptomes [28]. These lineage-pairs, representing different levels of divergence, were North and South populations of *C. rubrigularis* (N/S), Central and South *La. coggeri* (C/S), *La.* N/C, and *S. basiliscus* N/C. Additionally, we used comparative transcriptome data from *Le. bougainvillii*, *P. entrecasteauxii* and *P. pagenstecheri* (Griffith et al., unpublished data) for sequence divergence comparisons. To infer divergence history, we used the program 3s, which implements a coalescent model for isolation with migration [56]. 3s builds its inference across many genome loci sampled at one chromosome in each of the two focal lineages and an outgroup lineage. Previous work [57] has shown that with single samples per taxon, migration rates and ancestral population sizes are non-identifiable but that estimates of divergence time (tau) are robust, so we focus our comparisons on divergence time.

To generate haplotypes for our 3s runs, we first took the raw reads generated from AHE data collection, trimmed them for quality and adaptors Trimmomatic v0.16 [42], merged them using FLASH v1.0.2 [41], and removed sequence duplicates. For a given lineage-pair, we defined one reference assembly, to which we mapped both ingroup lineages and the outgroup. We mapped cleaned reads to the previously-generated assemblies using Bowtie2 [58] and called SNP variants using Samtools default parameters [59]. We phased variants using GATK ReadBackedPhasing [60] and filtered any variant and non-variant sites with coverage less than 5X. The resulting, coverage-filtered haplotypes were used in all downstream analyses.

We calculated divergence between the lineage-pairs using several metrics. First, we calculated nuclear divergence for both AHE and TM sequences [61]. Second, we used previously calculated estimates for divergence time in years, which has been estimated for all lineage-pairs but *P. entrecasteauxii* and *P. pagenstecheri* [29]. Briefly, we had previously inferred these divergence times by sequencing transcriptomes for each lineage (N = 5) in the lineage-pair, identifying variants in the untranslated regions of the lineage-pair, constructing the two-dimensional site-frequency spectrum (2D-SFS) for these variants, and then using dadi [62] to fit an isolation-with-migration model to the 2D-SFS. Third, we used 3s to infer tau in coalescent units. For each lineage-pair, we used all available loci (details on loci number, sequence length, and outgroups available in Additional file 2: Table S1), running each analysis ten times to ensure convergence across runs. We compared these estimates of divergence history to those obtained previously, expecting that, although the values should not be absolutely similar due to differences in units and mutation rates, divergence estimates should be correlated.

## Results

### Sequence data

From the original target set of 512 loci, we successfully captured and sequenced 415 across all samples including the outgroups. After applying coverage criteria, trimming all loci to the length of the second shortest sequence and removing all internal gapped characters, the mean locus length was 534 bp with a minimum and maximum lengths of 250 bp and 1458 bp, respectively (Note that loci with trimmed alignments shorter than 250 bp were removed). Of the 221,792 total characters, only 0.36% of the data was missing in any species.

**Figure 1:**
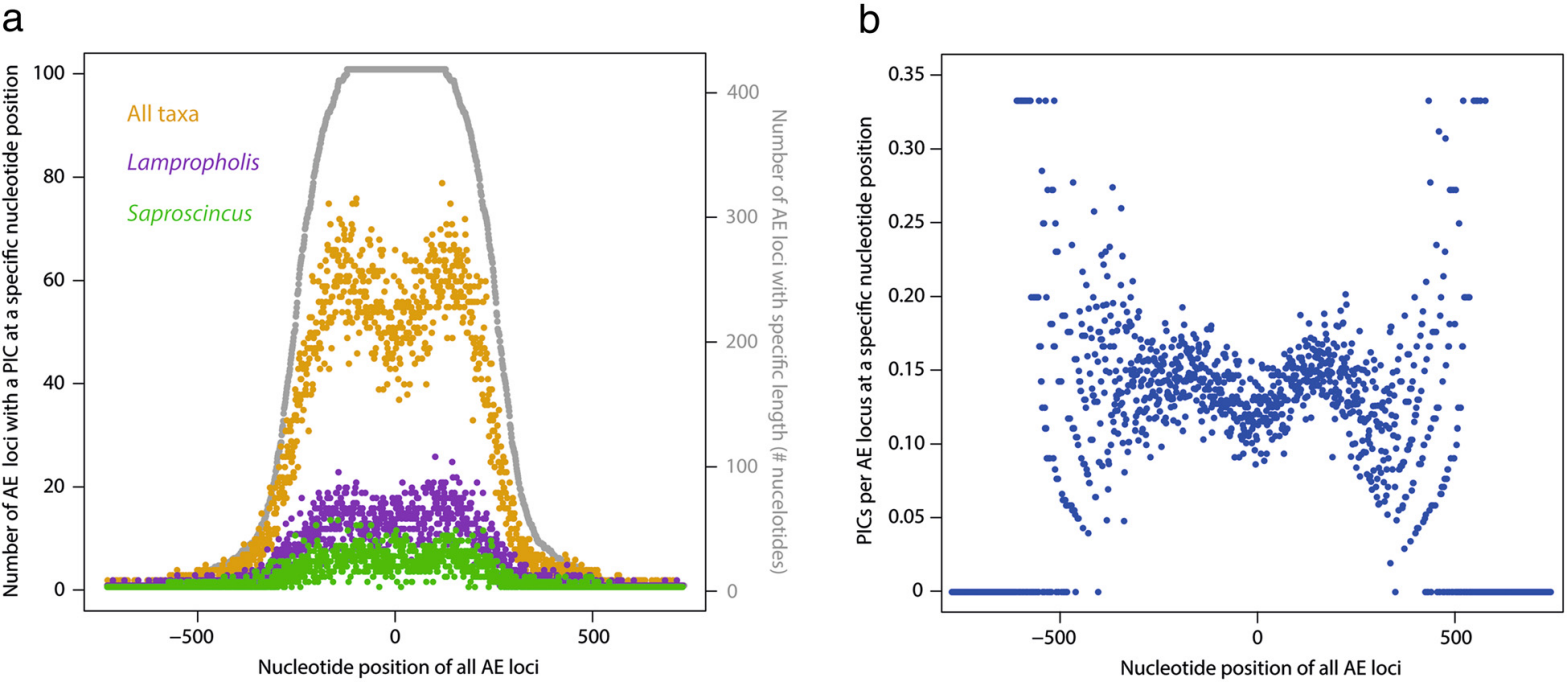
The distribution of parsimony-informative characters (PICs) for anchored hybrid enrichment (AHE) markers. **a**) The distribution of parsimony-informative characters (PICs) across the length of the 415 AHE loci for the full-taxon, *Lampropholis*, and *Saproscincus* data sets. Colored dots indicate how many total loci had at least one PIC at that nucleotide position. The zero position is the center of the anchor sequence for all loci. Grey dots indicate the distribution of locus length by plotting number of loci (right y-axis) with any nucleotide at that position. **b**) The distribution of PICs across the length of the 415 AHE loci in the full-taxon data set “normalized” by dividing the number of PICs at each nucleotide position by number of alignments of that length.

In the full 43 taxon data set, regions of the anchored-enriched (AHE) loci closer to the conserved anchor region (~ ± 120 bp) show slightly reduced numbers of PICs compared to more variable flanking regions (i.e. > ± 120 bp in Figure 1a). The decline in the number of PICs moving beyond ± 200 bp corresponds to a decrease in the number of loci of that size (i.e., > 400 bp). This pattern is similar, but less extreme, to that seen in UCE loci [7]. When normalized by the number of loci represented, the same trend is seen in the central ± 200 bp, with stochastic scatter beyond that (Figure 1b). The spatial distributions of variation within *Lampropholis* and within *Saproscincus* are similar, albeit less pronounced due to the fewer overall PICs. Complete details on AHE loci are available in Additional file 3.

For the TM analyses, we identified 10,847 exons of 7233 loci that met the initial filtering process. However, not all taxa were present for all loci. From this initial data set, we identified 1650 exons from 1648 genes that could be aligned across all ten taxa. These exons ranged in length from 101 to 3249 bp (mean = 337). Because all but two of our loci are single exons, we expect that phylogenetic error due to recombination is minimal. We refer to these 1650 exons as loci (rather than exons) to simplify comparative discussion with the AHE loci. Loci were trimmed so that all nucleotide positions are present for all genes (i.e., no missing data). Complete details on TM loci are available in Additional file 4.

### Phylogenetic information content and gene tree concordance

Across the full taxon AHE data set, and after excluding invariant loci, the mean number of PICs per locus was 52, but there was substantial variation across loci in information content with between 152 and 6 PICs per locus (Table 1). This variability is correlated with locus length (R^2^ = 0.19; P < 0.001). Within genera and species, mean information content was reduced, with *Niveoscincus* having the fewest PICs per locus (1.6; Table 1).

**Table 1:** **Summary statistics for the anchored-enriched (AHE) loci and transcriptome mining (TM) exons at different taxonomic levels and excluding non-informative loci**

			**Locus length**			**PICs**			**% identity**		
	**N samples**	**N loci**	**Max**	**Min**	**Mean**	**Max**	**Min**	**Mean**	**Max**	**Min**	**Mean**
Anchored-enriched (AHE)											
All samples	44	415	1458	250	534	152	6	52.1	99.6	92.1	96.5
*Carlia* ^1^	5	264	1458	250	541	36	1	2.3	99.0	94.1	99.0
*C. rubrigularis* ^1^	4	253	1458	250	540	36	1	2.2	99.9	94.5	99.4
*Lampropholis*	13	414	1458	250	534	44	1	15.0	99.7	95.0	98.0
*La. coggeri* ^1^	6	314	1458	250	543	13	1	3.1	99.9	97.8	99.4
*Niveoscincus* ^1^	5	207	1458	250	538	8	1	1.6	99.7	96.7	98.9
*Saproscincus*	9	412	1458	250	535	45	1	6.7	99.9	90.1	98.4
*S. basiliscus* ^1^	5	221	1458	250	555	13	1	2.1	99.9	95.6	99.5
Transcriptome mining (TM)											
All taxa	11	1650	3249	101	337	132	1	19.2	99.7	90.1	96.3
TM data pruned to number of AHE loci (highest PICs)											
All taxa	11	415	3249	163	617	152	23	41.3	99.1	95.3	90.1
TM data pruned to number of AHE loci (randomly sampled)											
All taxa	11	415	2480	103	337	120	1	19.2	99.6	90.8	96.3
AHE data pruned to TM taxon sampling											
All taxa	11	415	1458	250	534	96	1	25.4	99.7	91.8	96.3

^1^Phylogenetic analyses were not performed on these data sets.

The transcriptome mining (TM) data showed even stronger skew in information content (Table 1) and this was also correlated with locus length (R^2^ = 0.65; p < 0.001). Although TM produced more usable loci than AHE (1650 vs 415), both the maximum number of PICs and average PICs per locus are higher in the AHE loci (mean PIC_AHE_ = 52; mean PIC_TM_ = 19.2; Table 1). To compare the data sets more directly, we limited our AHE data set to the 11 taxa also included in the TM data set. Analyzing these data, we find the mean number of PICs per locus dropped from 52 to 25. Despite this reduction, PIC_AHE_ remains higher than PIC_TM_. We also trimmed the TM data to the 415 loci with the highest PICs to compare the most informative TM loci to the AHE loci. The mean number of PICs/locus in this data set is 41. To make a more direct comparison, we randomly selected 415 loci from the 1650 TM loci 10,000 times and mean number of PICs/locus (19) is identical to the entire 1650 locus data set, as expected.

#### Phylogenetic inference

**Figure 2:**
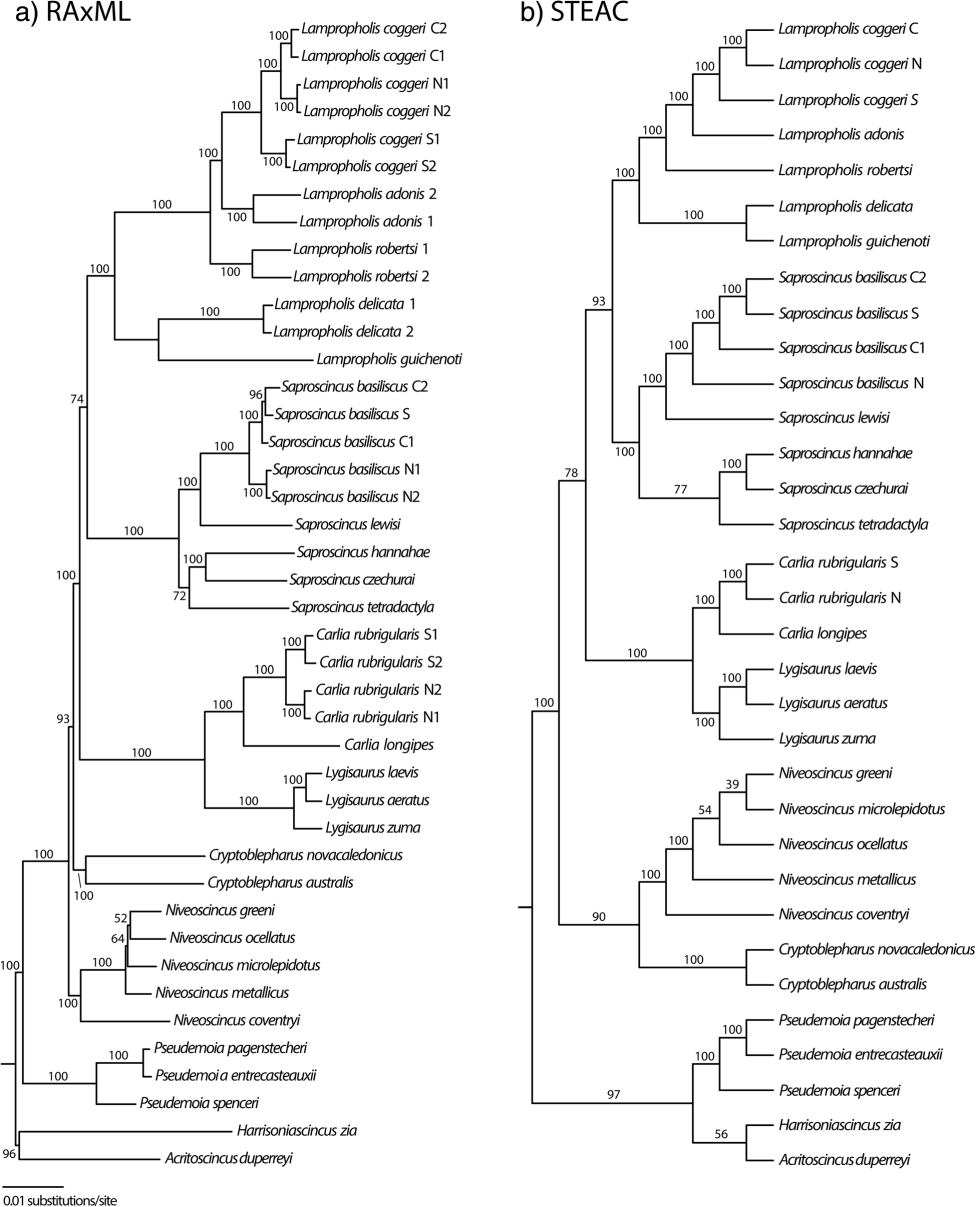
Results of the (**a**) RAxML maximum likelihood and (**b**) STEAC species tree analysis of 415 anchored enriched (AHE) loci for all taxa. Numbers above or below nodes indicate bootstrap proportions from 1000 pseudoreplicates. Outgroup not shown.

Analyses of the concatenated AHE loci using RAxML and using the STEAC species tree method were largely congruent, with high bootstrap support among phylogeographic lineages within species and among most congeners (except within *Niveoscincus*) (Figure 2). Monophyly of each genus with multiple species was strongly supported. By contrast, resolution among genera decreased towards the base of the tree, and there were two examples of strongly supported incongruence (bootstrap proportion [BP] > 90). In the RAxML analysis, the genus *Pseudemoia* forms a clade with all other Australian *Eugongylus* group skinks (BP = 100) exclusive of *Acritoscincus* (formerly *Bassiana*) and *Harrisoniascincus*; but it forms a clade with these two genera in the STEAC species tree (BP = 97). The genus *Cryptoblepharus* forms a clade with *Carlia*, *Lampropholis*, *Lygisaurus*, and *Saproscincus* (BP = 93) in the RAxML tree, whereas in the STEAC species tree this genus is sister to *Niveoscincus* (BP = 90).

Repeating the analyses at the congeneric to phylogeographic scale, there is no strongly supported incongruence amongst the concatenated RAxML tree and the STEAC species tree for either of the *Lampropholis* and *Saprosincus* datasets (Additional file 5: Figure S1), but clade support does differ at some nodes. Within *Lampropholis*, the RAxML and STEAC analyses strongly support the sister relationship of *La. coggeri* and *La. adonis.* Moreover, the relationships inferred among phylogeographic lineages within *La. coggeri* and within *S. basiliscus*, using either concatenation or species tree approaches accord with expectations from prior sequencing of multiple introns [24,28] (Figure 2).

**Figure 3:**
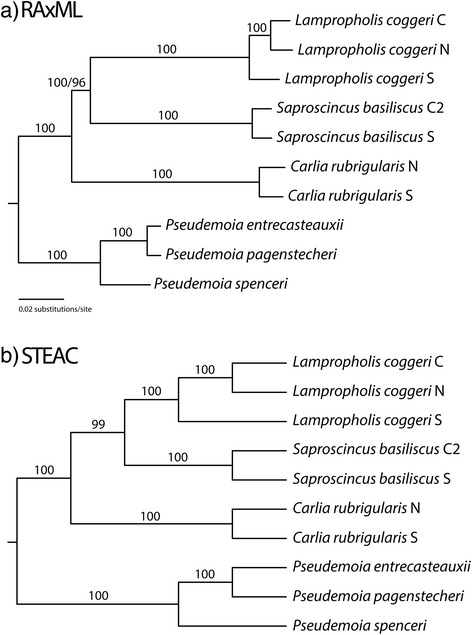
Results of the (**a**) RAxML and (**b**) STEAC analyses of all 1650 loci selected by our transcriptome mining (TM) analysis and the 415 anchored enriched (AHE) loci with taxa pruned the 10 ingroup and one outgroup taxa present in the TM data set. Numbers above the nodes in the RAxML tree indicate bootstrap proportions from 1000 pseudoreplicates. Clade support was identical between the TM and pruned AHE analyses, with the exception of the *Lampropholis* + *Saproscincus* clade that was supported by a bootstrap proportion of 100 and 96, respectively. We used PHYML maximum likelihood analyses to infer trees from 1000 bootstrap pseudoreplicates per locus. We used 1000 bootstrapped trees per locus (1,650,000 total trees) as input trees for the STEAC analysis, and numbers above or below the nodes indicate the proportion of times STEAC inferred that clade. Clade support was identical between the TM and pruned AHE analyses. Outgroup not shown.

To enable direct comparison between utility of AHE and TM at the phylogeographic scale, we trimmed the AHE data to just the taxa for which we had assembled transcriptomes. This included three lineages of *La. coggeri*, three closely related species of *Pseudemoia* and two lineages each for *S. basiliscus* and *C. rubrigularis* (Figure 3). Here, the inferred phylogenies were identical and completely resolved for both AHE and TM data sets.

### Divergence histories

**Figure 4:**
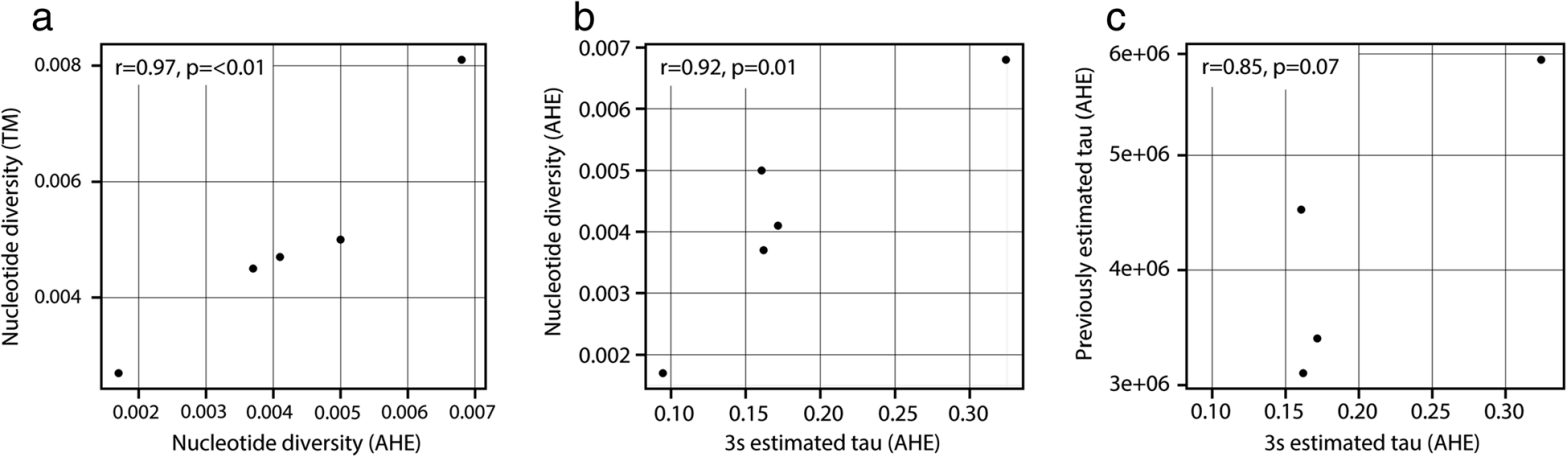
Correlations between different metrics for divergence, shown with one-tailed p-values. **a**) Correlation between nucleotide diversity at anchored enrichment (AHE) loci and transcriptome (TM) loci. **b**) Correlation between tau estimated by AHE loci using 3s and nucleotide diversity at AHE loci. **c**) Correlation between tau estimated by AHE loci using 3s and tau as previously estimated from population genomic data [31].

We inferred divergence for each of five lineage-pairs by comparing across nucleotide divergence, previously estimated using population sequencing, and the coalescent model implemented in 3s that samples a single chromosome per locus. Although AHE loci tended to have lower estimates than TM loci, nuclear divergence values at AHE and transcriptome loci were highly correlated (r = 0.97; P < 0.05; Figure 4a). Log-likelihood tests comparing models of divergence without gene flow (Model 0) and with gene flow (Models 1 & 2) in 3s supported divergence with gene flow for each of the five contacts (Additional file 2: Table S2). This contrasts with previous population-level inference [28], which supported divergence without gene flow in the four contacts (*C. rubrigularis* N/S*, La. coggeri* N/C and C/S*, S. basiliscus* N/C) tested. Tau estimates from a population history with gene flow were correlated with nuclear divergence at the same loci (r = 0.91; P < 0.05; Figure 4b), suggesting that gene flow during divergence was likely minimal. Tau estimates showed reasonable, though not significant, concordance with previously estimated divergence times (r = 0.85; P = 0.07; Figure 4c).

## Discussion

### Information content of phylogenomic data sets

We evaluated the phylogenetic information content of the anchored-enrichment capture approach (AHE) at multiple scales of divergence in Australian *Eugongylus* group skinks. By focusing on phylogeographic lineages that have been the focus of previous demographic inference (using multiple introns) and comparative transcriptome sequencing, we are able to gauge the performance of AHE loci against a broader sample of genes with varying rates of divergence.

### Performance of anchored hybrid enrichment and transcriptome mining

When using conserved targets such as those employed in AHE, we expected to see low phylogenetic resolution among phylogeographic lineages and higher resolution towards the base of the tree, but we did not uncover this pattern. Rather, relationships among phylogeographic lineages and congeneric species were well resolved, except the Tasmanian *Niveoscincus*. This genus aside, the overall good performance of AHE loci across closely related taxa reflects the presence of reasonable levels of diversity within, as well as adjacent to, the “conserved” AE probe regions. However, ambiguity remains for some deeper relationships in the *Eugongylus* group and phylogenies inferred from concatenation and species tree approaches strongly supported alternative placements of *Cryptoblepharus* and *Pseudemoia*. A potential explanation for this conflict and/or lack of resolution is that current phylogenetic methods cannot adequately model the complex processes of molecular evolution inherent in such large data sets. However, the generally short and poorly resolved branches among genera suggest a rapid radiation subsequent to colonization of Australia (as suggested for *Carlia* [30]), in which case there is strong potential for confounding of phylogenetic signal due to incomplete lineage sorting across gene trees [63,64]. Because concatenation does not account for conflict due to incomplete lineage sorting, the STEAC tree is our preferred hypothesis of phylogenetic relationships of the Australian *Eugongylus* group species. However, this hypothesis should be subject to further testing with additional nucleotide data and broader sampling of taxa.

#### Anchored-enriched loci

The key and positive result is that AHE loci provided mostly high phylogenetic resolution among closely-related species and phylogeographic lineages. This parallels the reported utility of another targeted enrichment approach – UCEs – for analyses of phylogeography [17]. Our primary test cases concerned phylogeographic lineages within three species of skinks from the Australian Wet Tropics rainforests (*Carlia rubrigularis, Lampropholis coggeri* and *Saproscincus basiliscus*) that have been the subject of multilocus analyses of phylogeography, historical demography and reproductive isolation in contact zones in the rainforests of north-east Australia [23-25,28,29]. The results of our analyses of the AHE loci add substantial support to previous analyses of 6–10 mostly intronic loci that found deep (~ Late Miocene to Pliocene) divergence and strong reproductive isolation between the two major lineages of *C. rubrigularis* and between the southern (“S”) and central (“C”) lineages of *La. coggeri*, and more recent divergence and weaker isolation between the C and northern (“N”) lineages of *La. coggeri* and the N and C lineages of *S. basiliscus*. The southern “S” lineage of *S. basiliscus* represents a case of strong discordance between deep mtDNA divergence and shallow nDNA separation from the adjacent (but now isolated) central “C” lineage; ABC coalescent modeling inferred that this was due to recent range expansion of C and introgression [28].

Further bolstering AHE loci’s utility for phylogeographic studies, we found that AHE loci yield sequence divergence estimates strongly correlated with those from TM data, and inferred divergence histories reasonably consistent with previous population-level studies of intron sequences. Often, researchers use rapidly evolving markers like introns to infer divergence history, with the assumption that such markers have substitution rates that allow us to investigate recent history [65]. Here, we show that AHE loci, despite largely being conserved across deep phylogenetic scales, are sufficiently informative that they can be used to estimate such divergence histories. That said, we note that our inference deviated from previous population genomic analyses. First, the AHE loci supported divergence with gene flow where previous inference did not, and second, the correlations, while on trend, are weak. These deviations could be due to differences in model assumptions between inference methods – i.e., how sensitive a method is to recombination, expectations for how gene flow should affect coalescent trees and times – and/or such discrepancies can arise simply from differences in bioinformatic pipelines [66]?

The phylogeny inferred from AHE loci in this study is identical to that obtained using all 1650 loci derived from transcriptome mining. This was the case even for *Saproscincus* lineages for which the information content of AHE loci and tree confidence factors were relatively low and noisy in relation to PIC scores. The stand-out exception is the poor resolution among closely-related species of Tasmanian *Niveoscincus*, evidently also a recent and rapid radiation (see also [67]). Ongoing improvements to the design of AHE targets, to increase locus length and information content by targeting adjacent exons and thus capturing intervening introns, will only serve to further increase their utility among closely related taxa (Lemmon and Lemmon, unpublished data).

Given the demonstrated strong performance of AHE loci for resolving a phylogeny at the phylogeographic scale of divergence and inferring divergence histories (and see also [17] for UCEs), along with the previous demonstrations of their utility at deeper phylogenetic scale (e.g., [9]), one could assume that these approaches will suffice for the vast majority of molecular systematics analyses. In particular, AHE loci exemplify *a priori* selection of loci to maximize both phylogenetic breadth of application and phylogenetic signal, both of which are emerging as key factors [1].

One caveat with the AHE (and UCE) approaches is the quality of alignments across non-coding regions adjacent to conserved anchor targets. By definition, conserved elements evolve slowly and therefore are expected to contribute few PICs at the shallow (e.g., phylogeographic) scale (Figure 1) as seen in UCE studies (Figure 2 in [17]). Although interspecific variation is lower close to the AHE target region compared to immediate flanking regions, there is nonetheless an intermediate level of variation as ~35% or more loci have at least one PIC close to the target region (Figure 1). As expected, the majority of PICs are from more variable flanking regions. On the other hand, sequencing coverage drops the farther from the anchor region, thereby increasing the possibility that variable sites are simply artifacts of sequencing error and low coverage (Figure 1). However, one could largely mitigate this problem by trimming flanking data so that all or most taxa share the same set of characters, and removing all gapped characters as we did in this study.

There will remain situations where broader sampling of exons is desirable, either to attempt to resolve short internodes, whether shallow or deep in the tree, or to estimate historical demography and divergence processes. Phylogenetic and species-delimitation analyses of genome-scale data remain constrained by computational limits, though there are promising developments in inference methods that use independent SNPs across thousands of loci [68,69].

#### Transcriptome mining

Transcriptome mining can potentially provide 1000s of loci with many PICs (see Supp Info 2) and can incorporate transcriptomes for phylogenetic analysis that were otherwise collected for other gene expression projects (e.g., [38]). Exons do have the advantages of more secure alignment via open reading frames, a broad spectrum of variability, and potential to link the frameworks of molecular evolution and phylogenetics [70]. Further, the development of custom exon capture systems from *de novo* assembled transcriptomes is now relatively routine [8] and will become all the more informative when paired with genomes from reasonably closely-related species. Depending on the application, thousands of exons sequenced can be analysed *en masse* (e.g. for population genomic analyses via site frequency spectra), or loci with the highest information content and most coherent modes of sequence evolution can be selected for phylogenetic analyses.

On the other hand, several aspects currently make TM less feasible for large-scale phylogenetics. First, transcriptome sequencing requires high quality RNA, preferably from fresh tissue or preserved in an RNA preservative and stored at ≤ −80C. This precludes ethanol-preserved tissues and older tissues that would otherwise preserve DNA. Second, transcriptome sequencing is still expensive when compared to AHE and exon-capture methods. Third, recombination between distant exons within single transcripts could violate assumptions of phylogenetic analyses, e.g., where all SNPs within a single locus are assumed to reflect a common gene tree history. This last phenomenon (termed a mixture tree in [1]) can be mitigated by using only a single exon from each gene.

### Phylogeny of the Australian *Eugongylus* group skinks

We inferred phylogenies of the Australian *Eugongylus* group lizards with generally high clade support. When compared to other studies including *Eugongylus* group taxa, several relationships match our expectations such as a clade including *Carlia* and *Lygisaurus* [30], the interrelationships of *Saproscincus basiliscus*, *S. czechurai*, *S. hannahae, S. lewisi*, and *S. tetradactyla* (the “northern lineage” of [71]), and a putative rapid radiation early in the Australian *Eugongylus* group’s history [21]. However, because there is no previous comprehensive molecular phylogenetic analysis of the group, most of our results are novel.

Despite using 415 loci (or subsets with the many PICs), some relationships remain unresolved or conflicting. The relationships of the Tasmanian species of *Niveoscincus* remain unknown. This lack of resolution likely represents a rapid radiation and that there is still insufficient amount of data (characters or taxa) to reconstruct the evolutionary history of these species. There is strong support for conflicting placements of both *Cryptoblepharus* and *Pseudemoia* between the concatenated data and STEAC analyses (Figures 3 and 4) reflecting the complex process of DNA evolution in these taxa as well. These conflicting or unresolved relationships continue to prohibit a complete understanding of placental evolution in *Niveoscincus* and *Pseudemoia* which exhibit a range of placental complexity from relatively simple placenta likely used for gas exchange and minimal maternal provisioning, to complex placentae with substantial maternal-to-embryo transfer [32,72]. Although TM performed well, it is not currently feasible to sequence transcriptomes for the remaining ~140 species of the group. Given the promising performance of AHE shown here, it is likely that further sampling across and within genera will do much to resolve the early phylogenetic history of the group.

## Conclusions

Our study demonstrates that, overall, anchored-enriched loci are informative at the intraspecific phylogeographic scale as well as deeper in the tree of life. Transcriptome mining provides a wealth of informative loci, but unlike hybrid enrichment methods, is less practical when scaling up to 100 s of taxa unless obtained via a capture approach [8]. In practice, we suggest a mixed approach that uses standard systems such as AHE or UCE to obtain initial results, and then, if warranted, more extensive capture of exons or anonymous loci for more detailed analyses of rapid radiations, divergence histories and population genomics (including for museum specimens [73]). Alternatively, mixed capture kits can be designed to span the scales of divergence to be studied for various projects. Design and testing of such capture systems containing mixtures of anchored loci (centered in exons) and anonymous loci (centered in randomly-chosen parts of the genome) are already underway in numerous animal and plant clades. These systems produce a spectrum of loci that are informative at the deep phylogenetic, phylogeographic, and population genetic scales.

## Availability of supporting data

The data sets and scripts supporting the results of this article are available in the Dryad repository, doi:10.5061/dryad.g4mj2.

## Additional files

Additional file 1:
**Localities of specimens used in this analysis.**
Additional file 2:
**Tables providing further information on demographic history inference.** **Table S1.** Information about the anchored enrichment (AHE) sequence alignments used for inferring divergence history with the program 3s. Assembly used refers to the reference assembly to which all reads in the trio (the lineage-pair and the outgroup) were mapped. **Table S2.** Log likelihoods for the three models fit to data in the 3s program. M0 assumes an isolation model without migration, and M1 and M2 assume an isolation model with migration. Significant likelihood ratio tests (LRT) critical values are 2.71 for comparing model M1 to M0 and 5.99 for comparing model M2 to M0. Additional file 3:
**Length, percent identity, and number of parsimony-informative characters (PICs) for the 415 anchored-enriched loci for the full taxon,** ***Lampropholis*** **only and** ***Saproscincus*** **only data sets.**
Additional file 4:
**Length, percent identity, and number of parsimony-informative characters (PICs) for the 1650 transcriptome mined exons for the full taxon,** ***Lampropholis*** **only, and** ***Saproscincus*** **only data sets.**
Additional file 5:
**Phylogenies for phylogeographic lineages within** ***Lampropholis*** **and** ***Saproscincus.*** (A) The concatenated RAxML tree for Lampropholis lineages. (B) The STEAC species tree for Lampropholis lineages. (C) The concatenated RAxML tree for Saproscincus lineages. (D) The STEAC species tree for Saproscincus lineages. Outgroups not shown.
