# Supplementary material for: Evaluating the performance of anchored hybrid enrichment at the tips of the tree of life: a phylogenetic analysis of Australian Eugongylus group scincid lizards
Source: BMC Evol Biol. 2015 Apr 11;15:62. doi: 10.1186/s12862-015-0318-0 (PMC4434831; doi:10.1186/s12862-015-0318-0)
Supplement: Additional file 2: — Tables providing further information on demographic history inference. Table S1. Information about the anchored enrichment (AHE) sequence alignments used for inferring divergence history with the program 3s. Assembly used refers to the reference assembly to which all reads in the trio (the lineage-pair and the outgroup) were mapped. Table S2. Log likelihoods for the three models fit to data in the 3s program. M0 assumes an isolation model without migration, and M1 and M2 assume an isolation model with migration. Significant likelihood ratio tests (LRT) critical values are 2.71 for comparing model M1 to M0 and 5.99 for comparing model M2 to M0. [file 12862_2015_318_MOESM2_ESM.docx]

**Table S1**. Information about the anchored enrichment (AE) sequence alignments used for inferring divergence history with the program 3s. Assembly used refers to the reference assembly to which all reads in the trio (the lineage-pair and the outgroup) were mapped.

| **Contact** | **Assembly used** | **Outgroup** | **N loci** | **Total Sequence Length** |
| --- | --- | --- | --- | --- |
| *C. rubrigularis* N/S | *C. rubrigularis* N | *La. coggeri* N | 479 | 526,093 |
| *La. coggeri* N/C | *La. coggeri* C | *La. coggeri* S | 466 | 367,888 |
| *La. coggeri* C/S | *La. coggeri* C | *La. coggeri* N | 466 | 367,888 |
| *P. entrecasteauxii* and *P. pagenstecheri* | *P. entrecasteauxii* | *P. spenceri* | 472 | 380,983 |
| *S. basiliscus* N/C | *S. basiliscus* C | *C. rubrigularis* N | 477 | 449,176 |

**Table S2.** Log likelihoods for the three models fit to data in the 3s program. M0 assumes an isolation model without migration, and M1 and M2 assume an isolation model with migration. Significant likelihood ratio tests (LRT) critical values are 2.71 for comparing model M1 to M0 and 5.99 for comparing model M2 to M0.

| **Contact** | **l M0** | **l M1** | **l M2** | **Best Model** | **Tau under best model (x100)** |
| --- | --- | --- | --- | --- | --- |
| *C. rubrigularis* N/S | -46997.9 | -46997.9 | -46992.0 | M2 | 0.161 |
| *La. coggeri* N/C | -39902.2 | -39902.2 | -39891.9 | M2 | 0.162 |
| *La. coggeri* C/S | -44205.3 | -44205.3 | -44169.3 | M2 | 0.324 |
| *P. entrecasteauxii* and *P. pagenstecheri* | -17441.2 | -17438.1 | -17437.8 | M2 | 0.095 |
| *S. basiliscus* N/C | -52026.3 | -52026.3 | -52009.9 | M2 | 0.172 |
