# Supplementary figures and images for: Evaluating the performance of anchored hybrid enrichment at the tips of the tree of life: a phylogenetic analysis of Australian Eugongylus group scincid lizards
Source: BMC Evol Biol. 2015 Apr 11;15:62. doi: 10.1186/s12862-015-0318-0 (PMC4434831; doi:10.1186/s12862-015-0318-0)

a) RAxML

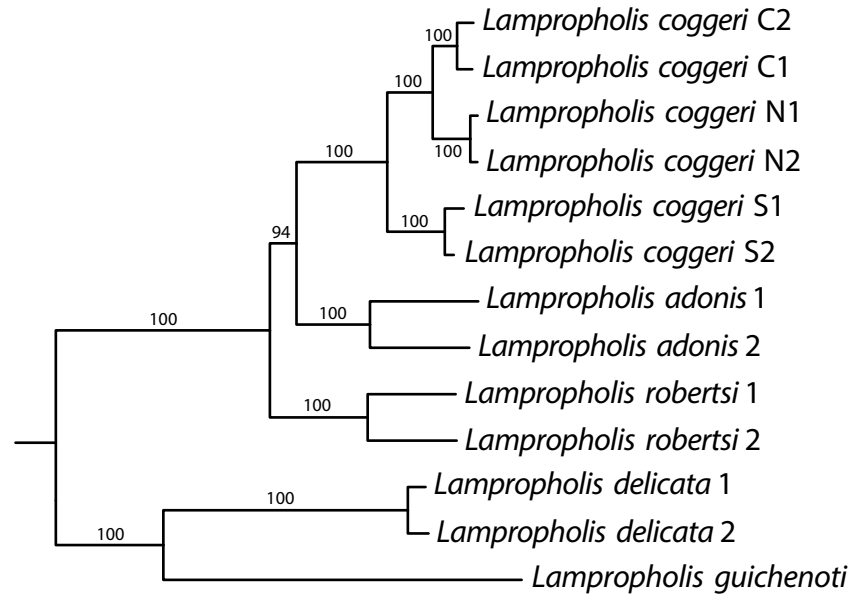

c) RAxML

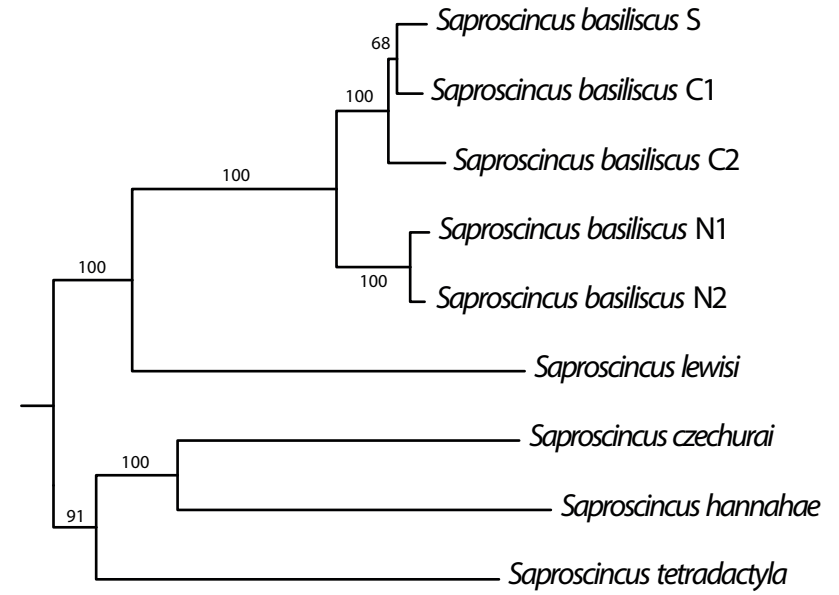

b) STEAC

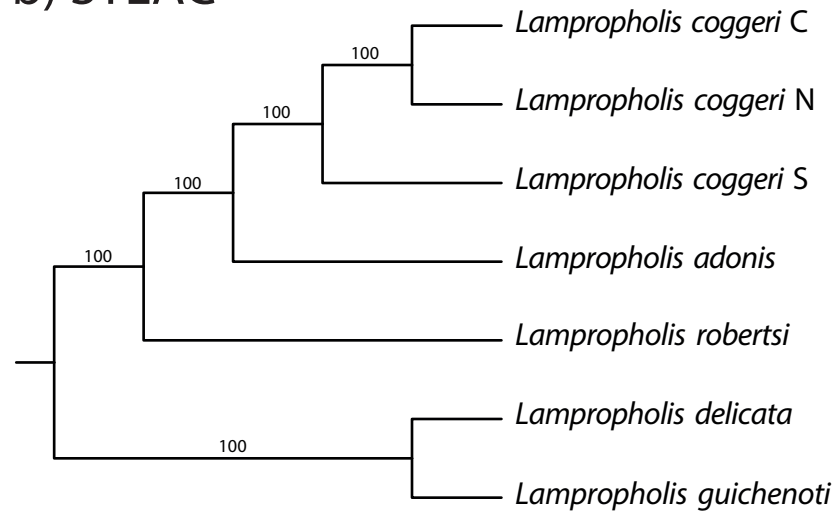

d) STEAC

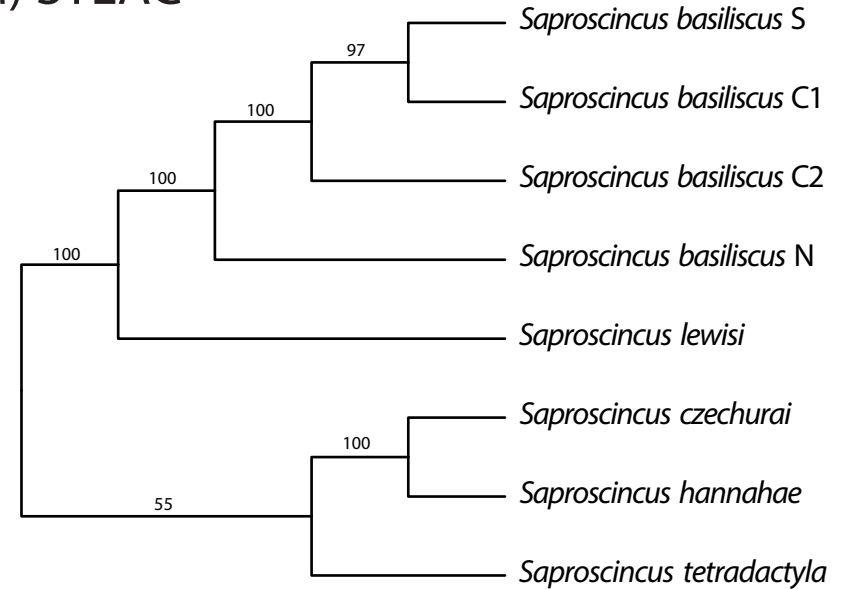

Supplement: Additional file 5: — Phylogenies for phylogeographic lineages within Lampropholis and Saproscincus. (A) The concatenated RAxML tree for Lampropholis lineages. (B) The STEAC species tree for Lampropholis lineages. (C) The concatenated RAxML tree for Saproscincus lineages. (D) The STEAC species tree for Saproscincus lineages. Outgroups not shown. [file 12862_2015_318_MOESM5_ESM.pdf]
